# Supplementary figures and images for: Endothelin B receptor inhibition rescues aging-dependent neuronal regenerative decline
Source: eLife. 2025 Sep 9;13:RP100217. doi: 10.7554/eLife.100217 (PMC12419800; doi:10.7554/eLife.100217)

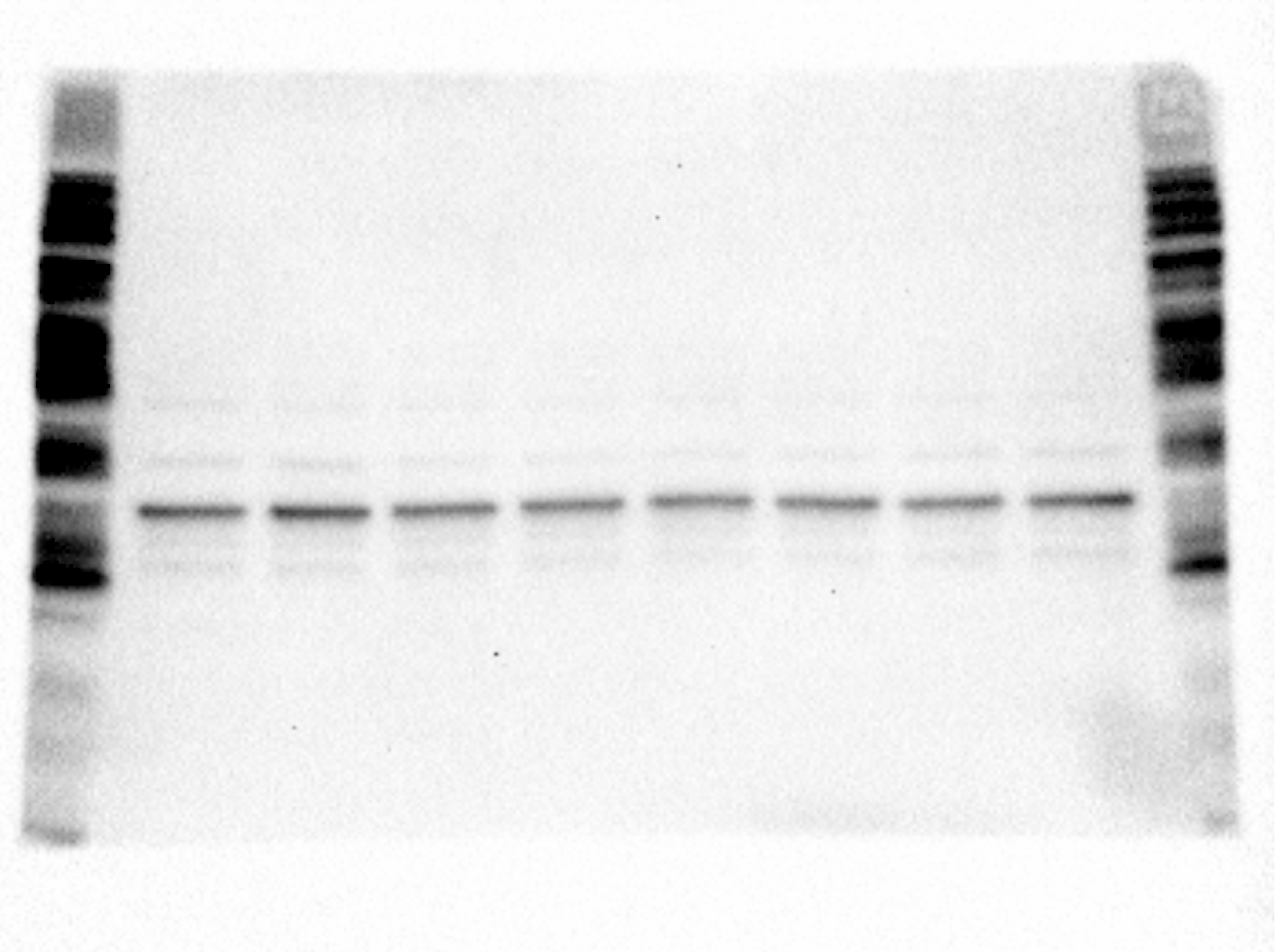

Supplement: Figure 3—figure supplement 1—source data 1. [file elife-100217-fig3-figsupp1-data1.zip › Figure 3-figure supplement 1-source data 1/M 60ug GAPDH Neuroscience 2022-04-04_18h15m41s_Exposure_60.0sec.tif]

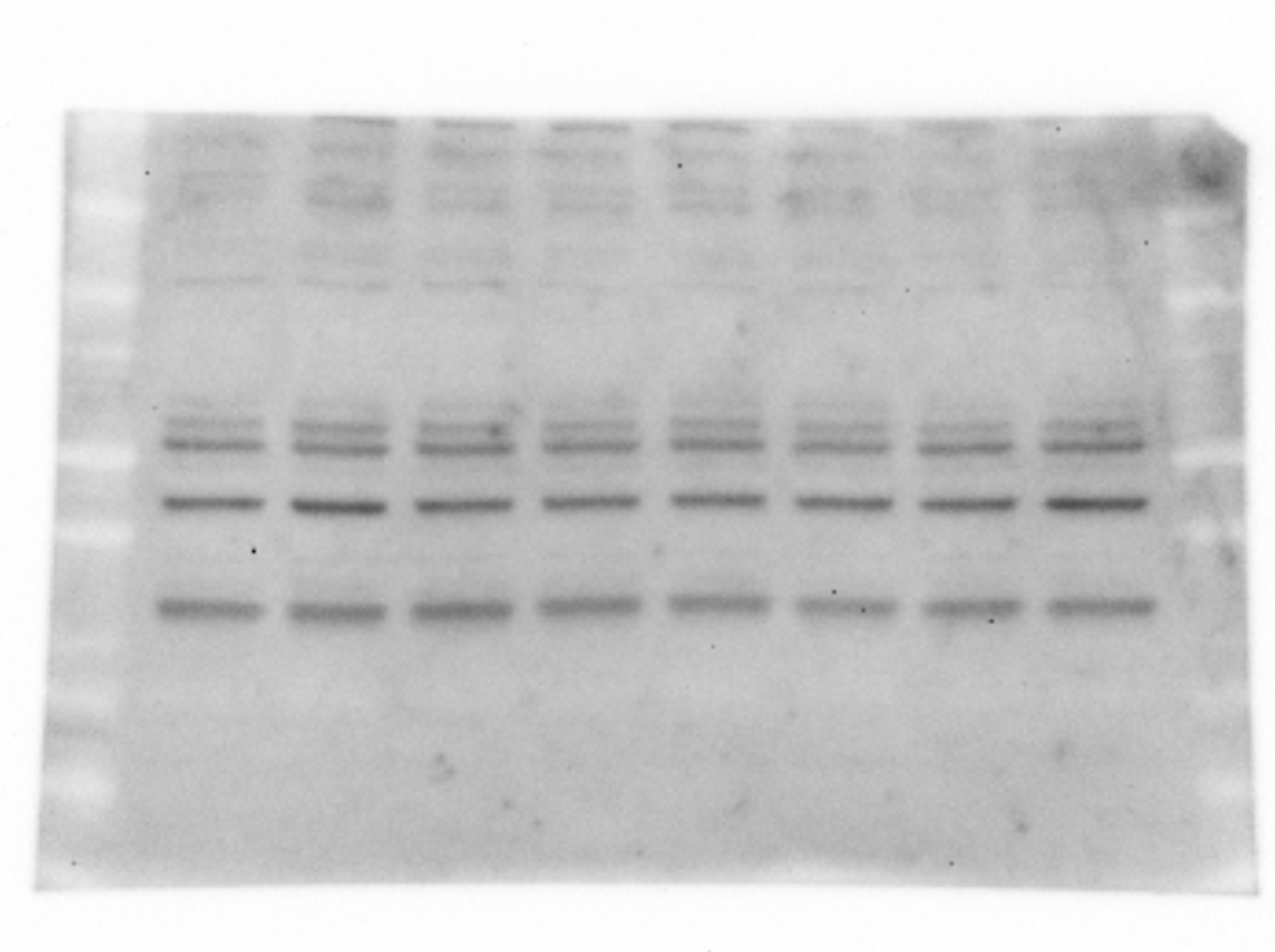

Supplement: Figure 3—figure supplement 1—source data 1. [file elife-100217-fig3-figsupp1-data1.zip › Figure 3-figure supplement 1-source data 1/M ETBR 50s Neuroscience 2022-03-25_15h37m49s.tif]

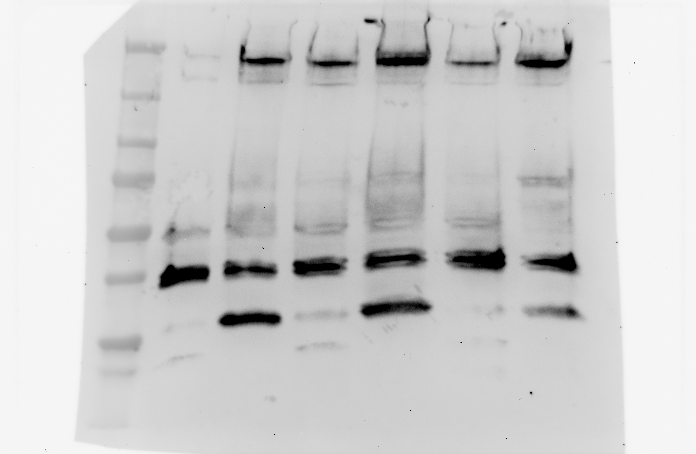

Supplement: Figure 4—figure supplement 1—source data 1. [file elife-100217-fig4-figsupp1-data1.zip › Figure 4- Supp Fig 1- Source Data 1/2025-04-10_11-18-07_Blot et1_Chemi_05_500s_view.png]

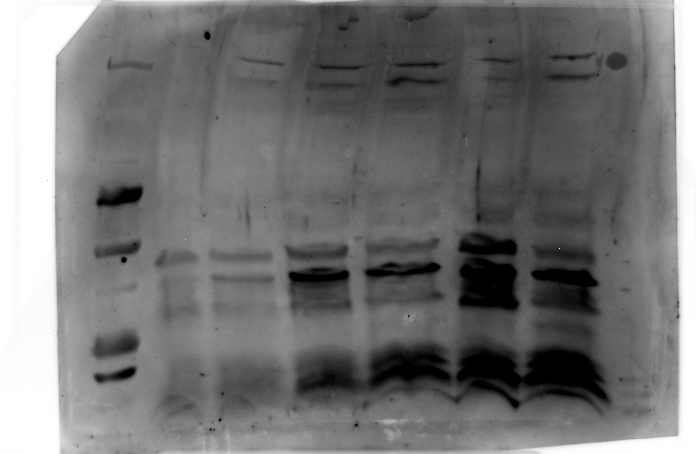

Supplement: Figure 4—figure supplement 1—source data 1. [file elife-100217-fig4-figsupp1-data1.zip › Figure 4- Supp Fig 1- Source Data 1/2025-04-10_11-58-36_Blot ETBR_Chemi_05_500s_view.png]

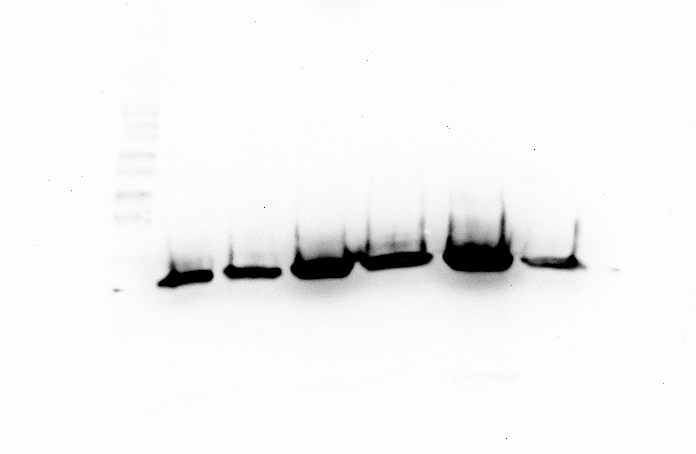

Supplement: Figure 4—figure supplement 1—source data 1. [file elife-100217-fig4-figsupp1-data1.zip › Figure 4- Supp Fig 1- Source Data 1/2025-04-18_12-52-55_Blot et1 gapdh _Chemi_03_180s_view.png]

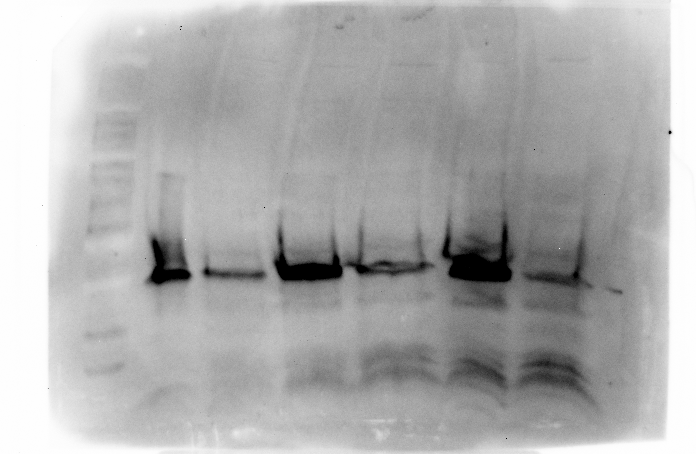

Supplement: Figure 4—figure supplement 1—source data 1. [file elife-100217-fig4-figsupp1-data1.zip › Figure 4- Supp Fig 1- Source Data 1/2025-04-18_13-06-44_Blot_Chemi_05_600s_view.png]

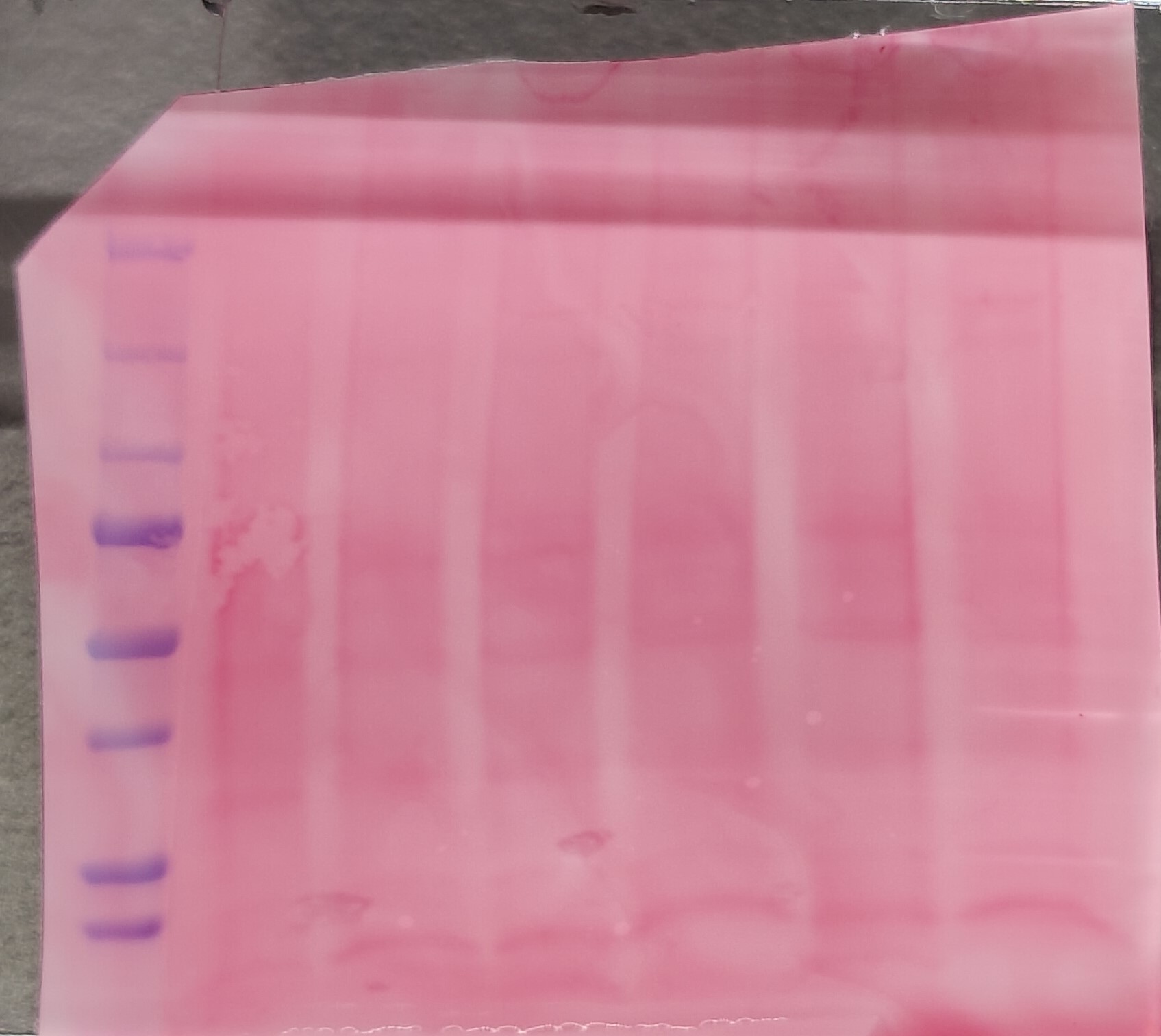

Supplement: Figure 4—figure supplement 1—source data 1. [file elife-100217-fig4-figsupp1-data1.zip › Figure 4- Supp Fig 1- Source Data 1/Ponceau- ET1.jpg]

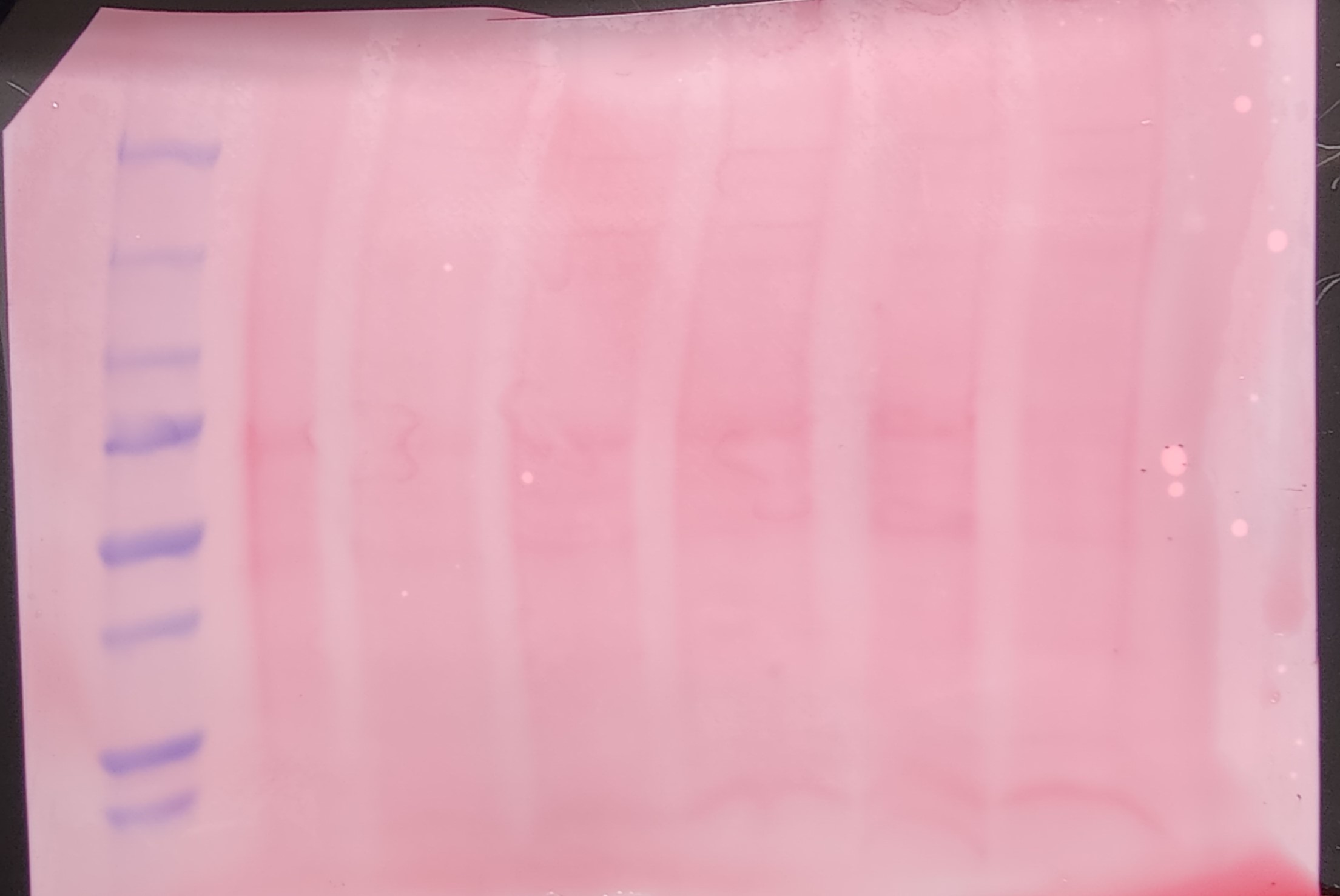

Supplement: Figure 4—figure supplement 1—source data 1. [file elife-100217-fig4-figsupp1-data1.zip › Figure 4- Supp Fig 1- Source Data 1/Ponceau- ETBR.jpg]
