# Supplementary material for: Endothelin B receptor inhibition rescues aging-dependent neuronal regenerative decline
Source: eLife. 2025 Sep 9;13:RP100217. doi: 10.7554/eLife.100217 (PMC12419800; doi:10.7554/eLife.100217)
Supplement: Figure 3—figure supplement 1—source data 2. [file elife-100217-fig3-figsupp1-data2.zip › Figure 3-figure supplement 1-source data 2/Fig 3- Fig Supp 1 Source Data 1 PDF containing original WB with relevant bands and treatments.pdf]

ETBR

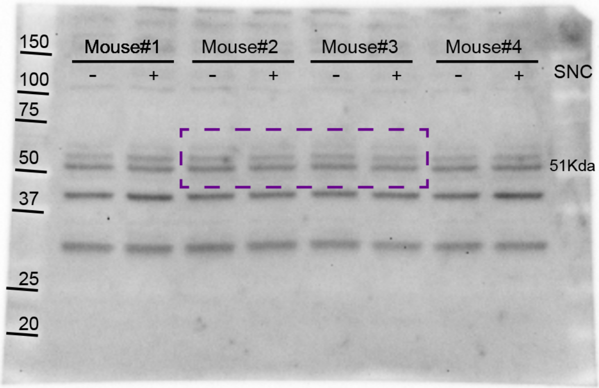
150
100
75
50
37
25
20
Mouse#1
Mouse#2
Mouse#3
Mouse#4
- + - + - + - +
SNC
51Kda

GAPDH

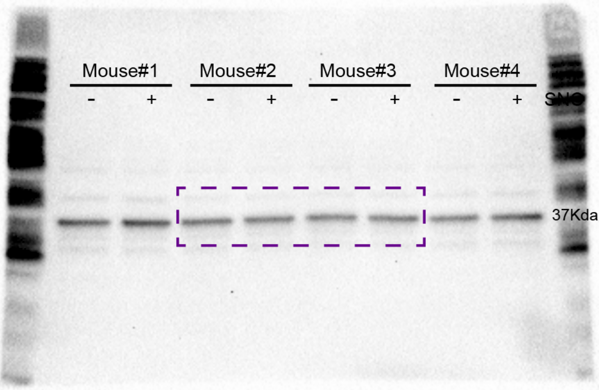
Mouse#1
Mouse#2
Mouse#3
Mouse#4
- + - + - + - +
SNC
37Kda
