# Supplementary material for: Endothelin B receptor inhibition rescues aging-dependent neuronal regenerative decline
Source: eLife. 2025 Sep 9;13:RP100217. doi: 10.7554/eLife.100217 (PMC12419800; doi:10.7554/eLife.100217)

ET1 Blots

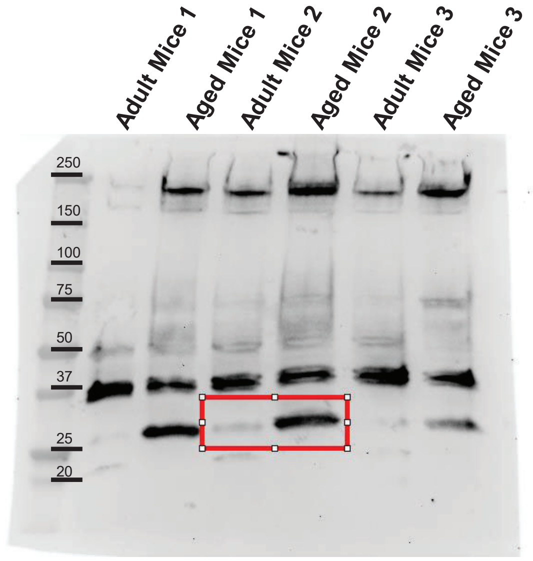

Etbr Blots

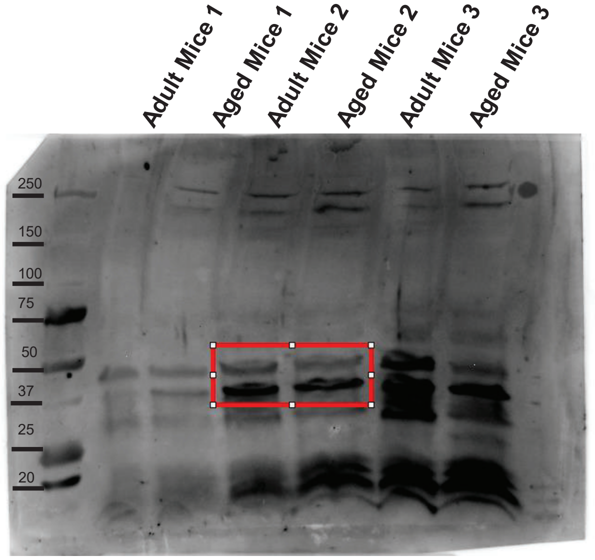

GAPDH of ET1 Blots

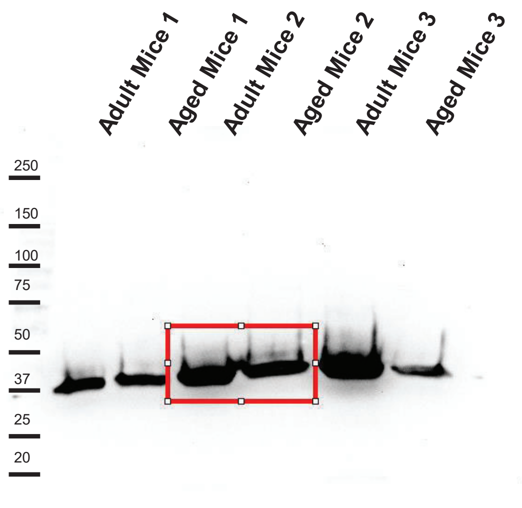

GAPDH of ETBR Blots

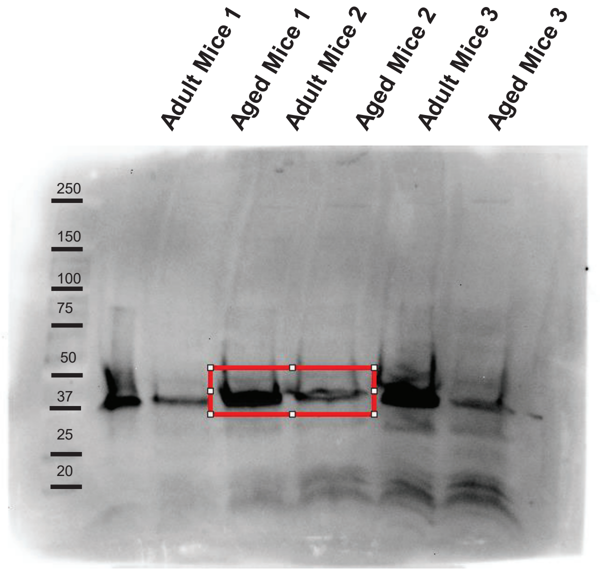

Ponceau of ET1 Blots

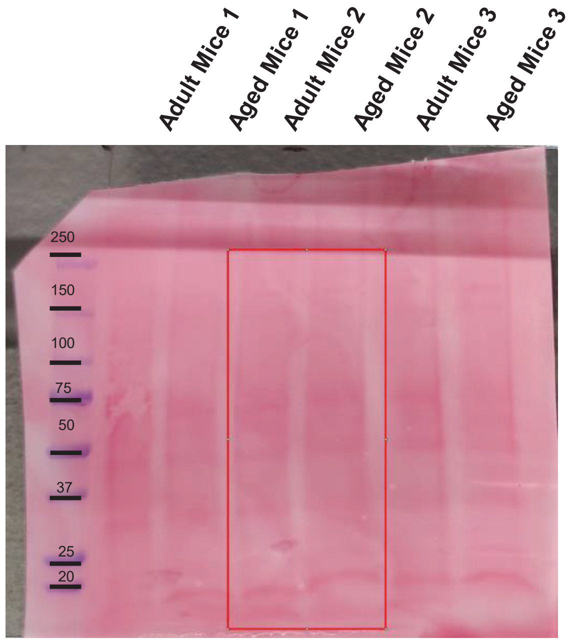

Ponceau of Etbr Blots

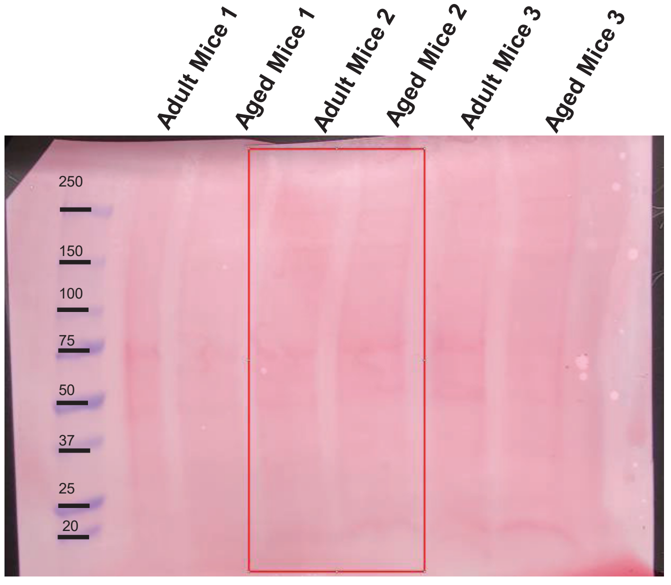

Supplement: Figure 4—figure supplement 1—source data 2. [file elife-100217-fig4-figsupp1-data2.zip › Fig 4- Fig Supp 1 Source Data 2/Fig 4- Fig Supp 1 Source Data 1 PDF containing original WB with relevant bands and treatments.pdf]
